# Medical Society of London

**Published:** 1847-10

**Authors:** 


MEDICAL SOCIETY OF LONDON.

Dr. Marshall Hall read a paper on the *Convulsive Affections of Infants and Children.*

The author began by alluding to the dangers attendant on infantile convulsions, to its consequences to mind, limb, and life, and to the possibility of idiocy, or liability to epilepsy, being its result. He then made reference to the causes, forms, and effects of such convulsions, and the mode by which they are induced; and then proceeded more particularly to consider them. He dwelt especially on—

1. *The terms employed* to designate certain forms and symptoms of them; and on one especially, laryngismus stridulus, which the author contended was no more a disease than cough was a disease, or "any other symptom of disease was a distinct disease." He said, that laryngismus was not always stridulous, but depended on the same causes, whether it was or was not so; the most dangerous forms of it were those which were noiseless. He would associate this symptom, which was certainly one of great peculiarity and danger often, with contraction of the hand, which he would call chirismus, and with that of the foot, which he would style podismus; the term sphincterismus, too, might be applied to spasm of the sphincter ani, or neck of the bladder. "Let the termination in *ismus* be used only to designate a symptom, and that of a purely nervous or convulsive character."

2. *The predisposition* to convulsive affections, and laryngismus more especially, was very marked. The latter had been known to affect a whole family. The cause of such predisposition is obscure: was it hereditary? was it the effect of locality, or emanations from the soil?

3. *The causes.*—No irritation of the cerebrum or cerebellum could *immediately* produce muscular spasm, as experiment had shown again and again. But irritation of the membranes of the brain might excite it, as appeared from an experiment which he had performed, and recently detailed. Irritation of the medulla oblongata, or medulla spinalis, produced the most frightful spasms. The incident nerves, when affected at their origin in the cutaneous, mucous, or other tissues, were the most frequent source of the attacks. The condition of the gums in teething, gastric or intestinal disorder; matters retained in the lower part of the alimentary canal; the atmosphere itself, especially when north, east, or north-east winds prevailed; perhaps certain vapours;—these were all insisted on as being intimately connected with the production of convulsion, or that form of it called laryngismus. Strabismus, or the spasmodic condition of the hand or foot, might arise from teething, &c.; but the larynx was very apt to be affected by the north or east winds, or other conditions of the atmosphere. He also associated laryngismus stridulus with undue excitability of the spinal centre: when it seemed got rid of, it was very apt to recur. Hence the precaution of persevering with remedies longer than would otherwise be necessary.

4. *The influence of sleep.*—He alluded to the frequent occurrence of convulsions at this period, chiefly epilepsy. There was congestion of the nervous centres then; probably unusual excitability of them. Altogether, it produced a state favourable to convulsive seizures.

5. *Cerebral diseases.*—On this the author forcibly insisted. He referred to the consequences of inflammation, tubercular granulation or tumour, and effusion at the base of the brain; and also to the congestion of pertussis.

6. *Excited reflex actions.*—By far the greater number of convulsions were of a reflex nature. Laryngismus was most effectually avoided by removing every exciting cause of reflex action. He would chiefly guard against four causes of such action: first, irritation of the trifacial nerve, which took place in teething; second, that of the pneumogastric nerve; third, irritation of the spinal nerves; and fourth, the effects of the atmosphere upon the larynx, under certain circumstances. The organs affected in a convulsive seizure were precisely those which its pathology would lead us to expect—the larynx, the sphincters, &c. The author then called the attention of the society to certain bronchitic, hepatic, and renal symptoms, and to the condition of the urine,—points which needed further investigation. He then dwelt on the effect of—

7. *Emotion, passion,* and showed how great and important was the part which they played in the affections he was treating of. He enforced the necessity of bearing them in mind fully in certain cases; he showed that they often constituted the real and only objection to the use of the gum-lancet, which consequently should always be cautiously employed.

8. The effects of augmented excitability were insisted on. States of the nervous system, induced by mild electricity, were compared with those occasioned by disease. The results of increase of excitability were entered into—irritants then acted, which at other times would be inert. A change in the direction of the wind, even, was not without bad consequences. Strychnia induced a species of laryngismus. Emotion, hysteria, epilepsy, tetanus, hydrophobia, all affected the larynx in a special manner.

The author next described those affections of the cerebrum which were consequent on convulsions,—the congestion, the effusion, the occasional paralysis, the risk of idiocy, &c. He then passed on to the question of sudden dissolution, demonstrating how difficult it was to forsee it often, and stating how frequently it happened when the patient appeared in progress to recovery. It was the result of common asphyxia, but not rarely of what he had called secondary asphyxia, which he believed was closely dependent on the blood of the coronary arteries being unduly arterialized. The remedies of asphyxia should be enforced promptly in such cases of sudden death.

Some observations were then made on the diagnosis of convulsions, in which the transient, or permanent, or complicated character of symptoms, as the case might be, were all pointed out as modes of assistance in conducting the inquiry. The author drew attention to the post mortem appearances, which varied as the disease was centric or eccentric, or according to the mode of death. There might be the results of inflammation within the cranium, or nothing found whatever but the appearances proper to asphyxia. Lastly, he made some practical observations upon prevention and treatment; as to the latter insisting on an accurate diagnosis as an indispensable preliminary, on a due attention to the complications of the affection, on the necessity of bearing in mind all the varied forms of irritation, and applying the appropriate remedies without delay, on having regard to the state of the patient during the time of sleep, on protecting it from cold air, &c. And if he had shown the application of the physiology of the nervous system to its pathology, he had gained the object which he had in view in bringing the subject before the society.

Mr. Hird considered that the profession were much indebted to Dr. Hall for his researches on the subject of infantile convulsions, and for his explanation in respect to those cases in which the brain was involved in the cause, and where it was not. He agreed generally in the views of the author, but should be afraid to lance the gums so freely and so often as Dr. Hall had recommended in some of his published papers. In the other plans of treatment recommended he fully concurred.

Mr. Barlow agreed fully with Dr. M. Hall as to the ill consequences of cold in laryngismus stridulus. In some cases, a keen wind was certain to bring on the paroxysm. In a case related by Dr. Hugh Ley, the first attack was produced by the application of cold to the head. He thought no one could contradict the correctness of the view which had been taken of the causes of the disease. He believed by far the larger number of cases were eccentric in their origin, and that depleting measures should never be used without much caution. Irritation of the trunk of the nervus vagus produced reflex action, contrarily to what happened in the nerves proceeding to the limbs; and he thought that in disease, spasm of the glottis, either with or without crowing, might occasionally be brought on by affections of the trunk of this nerve, giving rise either to direct or reflex closure of the glottis. In a case where Sir Astley Cooper tied the carotid artery, inflammation and suppuration extended upwards in the course of the nervus vagus, and there was a cough, like that of hooping-cough. Sir Henry Marsh, in his instructive paper on spasm of the glottis, had suggested irritation of the origin of the pneumogastric as a cause of the affection; but the state of parts far remote from the nervous centres was mostly at fault. He had never been able to associate enlargement of the bronchial or cervical glands with the disease by way of cause and effect, as Dr. Hugh Ley had done in a work which would ever be consulted for its abundant information in regard to the malady. In two cases he (Mr. Barlow) had found it connected with hydrocephalus; in another, which he had examined after death, with bronchitis; in a fourth, which was fatal, he thought that the last paroxysm had depended on over feeding. In the country—he meant the country properly speaking—the disease was acknowledged to be rare; and even in the crowded districts of towns he thought it rarer than was supposed. Out of 6879 patients who had been admitted at the Children's Infirmary, since January, 1st, 1846, there were only seven cases reported of this disease. In three cases he had observed the paroxysm produced by the act of drinking—a fact of interest, viewed as an addition to those phenomena which connected laryngismus with the convulsive actions, of which it was certainly one. He would ask Dr. Hall if he had observed this fact.

Dr. Theophilus Thompson remarked, that in the majority of obstinate cases of laryngismus stridulus, hydrocephalus was either present, or threatened to develope itself. Sometimes convulsions were the result of simple irritation; in other instances they originated in inflammation.

Dr. Clutterbuck thought that the brain was always involved in cases of convulsions, and that it suffered at these times from inflammation. The brain was a complicated organ, and various parts of it performed various functions. He agreed in the treatment recommended by the author.

Dr. Reid did not find that dampness of the atmosphere was a cause of laryngismus; on the contrary, the affection was rare in damp localities. He had some doubts respecting the prejudicial influence of a north-east wind in these cases, and mentioned two instances in which it had no such bad effects. He had never seen a case during the time the infant was suckling.—*Dublin Medical Press.*

